# Aquaporins 1, 3 and 8 expression in irritable bowel syndrome rats’ colon via NF-κB pathway

**DOI:** 10.18632/oncotarget.17565

**Published:** 2017-05-02

**Authors:** Guanqun Chao, Shuo Zhang

**Affiliations:** ^1^ Department of Family Medicine, Sir Run Run Shaw Hospital, Zhejiang University, Hangzhou, China; ^2^ Department of Gastroenterology, The First Affiliated Hospital, Zhejiang Chinese Medical University, Hangzhou, China

**Keywords:** irritable bowel syndrome, aquaporins, NF-κB

## Abstract

**Objective:**

Our research was to detect the expression of aquaporins. NF-κB in Irritable bowel syndrome (IBS) rat models’ colon so as to find novel pathogenesisof IBS.

**Results:**

The expression of AQP1, AQP3, and AQP8 of IBS model group was down-regulated while NF-κB p65 was up-regulated comparing with control group (*p* < 0.05), and the expression of AQP1, AQP3, and AQP8 of inhibitor group was up-regulated while NF-κB p65 was down-regulated comparing with IBS model group (*p* < 0.05).

**Materials and Methods:**

18 adult female SD big rats were divided into three groups:the rats in control group were normal rats, the rats in IBS model group and the rats of inhibitor group were injected with the inhibitor of NF-κB (PDTC). Immunohistochemical technique and western blot were performed to detect the expression of AQP1, AQP3, AQP8 and NF-κB p65. RT-PCR was performed to detect the expression of AQP1, AQP3, and AQP8.

**Conclusions:**

Liquid water metabolic abnormalities and intestine permeability alteration might be the mechanism of IBS by down-regulating AQP1, AQP3 and AQP8 via NF-κB pathway.

## INTRODUCTION

Irritable bowel syndrome (IBS) is a common functional gastrointestinal disorder, appearing as complex symptoms such as abdominal pain/discomfort [1]. As a chronic biopsychological disorder, IBS is characterized by altered bowel habits excluding organic disorders. Except for the astrointestinal motility abnormality and distorted visceral perception of sensation, IBS is also connected with several gastrointestinal and extraintestinal manifestations [2]. Although IBS is one of the most common disease, the etiology of the disease remains unknown. Several physical and psychological factors, such as stress, anxiety, and abnormal attitudes towards illness, are known to contribute to IBS's pathogenesis [3]. Nowadays the worldwide prevalence of IBS ranges from 3% to 22% of the population [4] and in America, IBS affects 15% of the population [5]. Current evidence figures out that the diagnosis of IBS is still according to Rome IV and the actual quantity of the presence of such underlying cause of IBS, however, IBS's pathogenesis remains unknown. Therefore, the objective of our research was to find novel pathogenesis so as to contribute to the treatment of IBS.

In the human body, aquaporins (AQPs) are considered play important roles in the water transport system [6]. There are currently 13 types of AQP, AQP0 through AQP12, which are expressed in various organs. Taking intestinal tract as example, AQP1, AQP3, AQP4, AQP7, AQP8, AQP9, and AQP10 has been found expressed in the colon, which affects fecal water content [7–9]. It has been improved that the functions of AQPs in the stomach and intestine physiology may involve water transfer, gastric juice secretion, barrier function, as well as absorption and secretion of water and even small solutes through the epithelium [7, 10]. As the gastrointestinal tract is the major organ for water transport which is only secondary to kidney, IBS might have some change in water transport through AQPs. It has been found that AQP1,AQP3,AQP8 were abundantly expressed in rat intestinal epithelial cells, and were also expressed in the human colon, as a result of this, we chose to study these three AQPs [11–12].

The nuclear factor-kappa B (NF-B), which can be activated by a variety of stimuli such as virus infection, activation of kinases and oncogenes, overproduction of cytokines, and dysregulation of cell surface receptors, plays an important role in the process of immune responses and inflammation [13]. It has been reported that the expression of AQP5 mRNA in the parotid gland was down-regulated after LPS inducing, which is mediated via transcription factors NF-kB and p-c-Jun/c-Fos [14]. Another research reported that NF-kB activation is of great importance for the down-regulation of AQP2 channel and vasopressin receptor expression during sepsis [15]. As a result of the mechanism of IBS include immune responses and inflammation, we speculated that NF-kB pathway might play an important role in IBS. Our research was to detect the expression of AQPs in IBS rat models and to detect the expression of AQPs with the inhibitor of NF-kB so as to elucidate the mechanism of IBS.

## RESULTS

### The change of the model

After completing the models, we found that there was no change of behavior. Rats of IBS model group (including model group and inhibitor group before injection) expressed excessive reaction when they were frightened or they were performed by intragastric administration. There was also no change of their stool.

### Model authentication

The rectum effusion amount of the model rats(including model group and inhibitor group before injection) (0.88 ± 0.16 ml) were lower than the control group (1.42 ± 0.11 ml), the difference had statistical signifcance (*P <* 0.01), which suggested that model building method was successful (Table 1).

**Table 1:** The water injection rate when AWR score was 3

	Control group (ml)	IBS Model group (ml)
The water injection rate	1.467 ± 0.054	0.833 ± 0.143

*P <* 0.05 compare with control group.

### The expression of AQP1 in the colon by immunohistochemical technique

### AQP1 cell immunochemical staining

**Figure 1:**
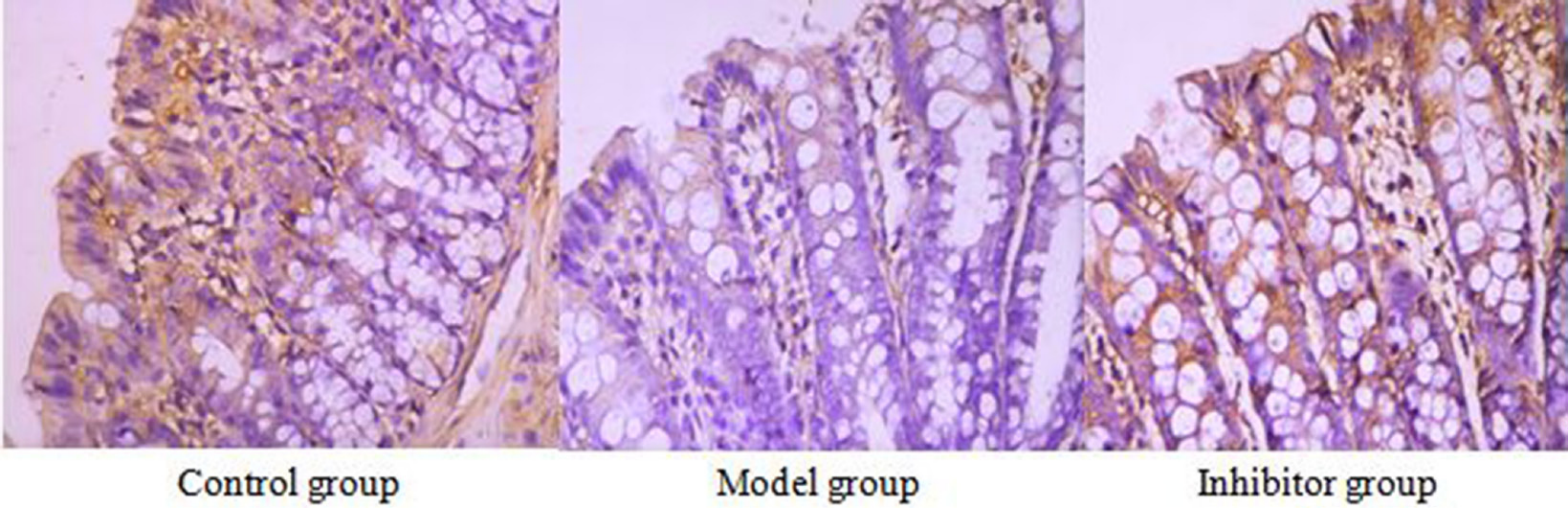
The expression of AQP1 in the colon by immunohistochemical technique (*400) AQP1 positive reaction material presents brown, the cell membrane and cytoplasm were dyed

AQP1 positive reaction material presents brown, the cell membrane and cytoplasm were dyed, and the negative control was not dyed (Figure 1).

### The expression of AQP1 comparing among the different group

The positive cell numbers of model group was less than the control group (*P <* 0.05), the positive cell numbers of inhibitor group was more than model group (*P <* 0.05), and the positive cell numbers of inhibitor group was more than control group(*P <* 0.05) (Table 2).

**Table 2:** The expressions of AQP1, AQP3, AQP8 and NF-kB p65 in the colon by immunohistochemical technique

	Control group	Model group	Inhibitor group
**AQP1**	1.348 ± 0.190	0.3031 ± 0.048 *^a^*	0.675 ± 0.077 *^b^*
**AQP3**	2.011 ± 0.178	0.957 ± 0.138 *^a^*	1.859 ± 0.176 *^b^*
**AQP8**	2.401 ± 0.216	0.609 ± 0.120 *^a^*	1.847 ± 0.190*^b^*
**P65**	0.204 ± 0.022	2.129 ± 0.263 *^a^*	0.425 ± 0.054 *^b^*

*^a^P <* 0.05 compare with control group; *^b^P <* 0.05 compared with model group.

### The expression of AQP3 in the colon by immunohistochemical technique

### AQP3 cell immunochemical staining

**Figure 2:**
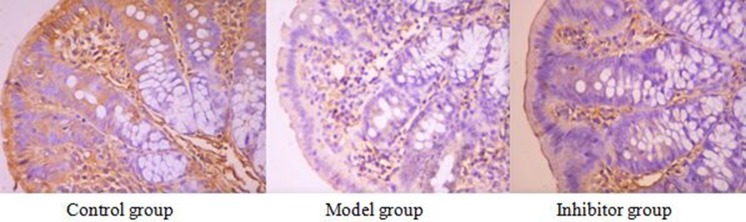
The expression of AQP3 in the colon by immunohistochemical technique (*400) AQP3 positive reaction material presents brown, the cell membrane and cytoplasm were dyed

AQP3 positive reaction material presents brown, the cell membrane and cytoplasm were dyed, and the negative control was not dyed. (Figure 2).

### The expression of AQP3 comparing among the different group

The positive cell numbers of model group was less than the control group (*P <* 0.05), and the positive cell numbers of inhibitor group was more than model group (*P <* 0.05), nevertheless, there was no significant divergence between control group and inhibitor group (*p >* 0.05) (Table 2).

### The expression of AQP8 in the colon by immunohistochemical technique

### AQP8 cell immunochemical staining

**Figure 3:**
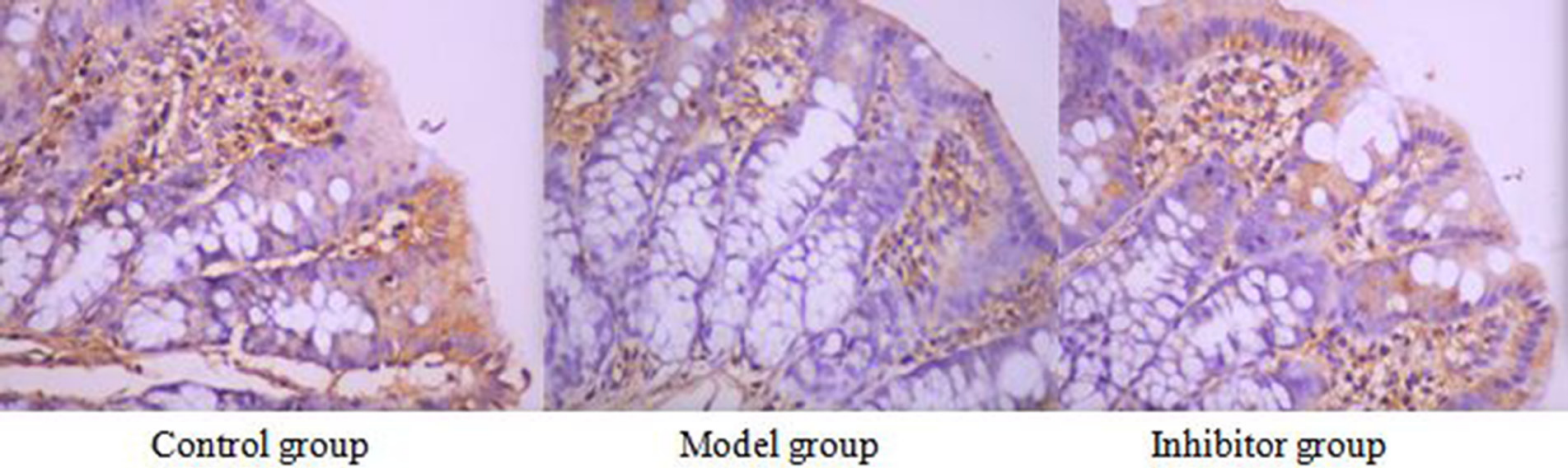
The expression of AQP8 in the colon by immunohistochemical technique (*400) AQP8 positive reaction material presents brown, the cell membrane and cytoplasm were dyed

AQP8 positive reaction material presents brown, the cell membrane and cytoplasm were dyed, and the negative control was not dyed. (Figure 3)

### The expression of AQP8 comparing among the different group

The positive cell numbers of model group was less than the control group (*P <* 0.05), the positive cell numbers of inhibitor group was more than model group (*P <* 0.05), and the positive cell numbers of inhibitor group was more than control group(*P <* 0.05). (Table 2)

### The expression of NF-kB p65 in the colon by immunohistochemical technique

### NF-kB p65 cell immunochemical staining

**Figure 4:**
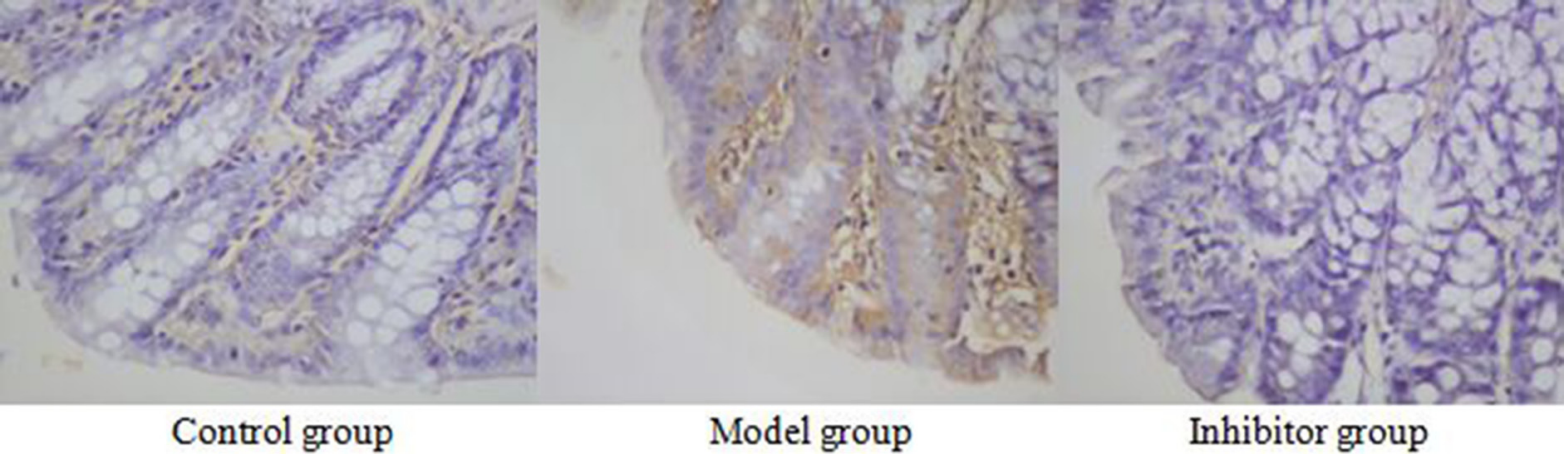
The expression of NF-κB p65 in the colon by immunohistochemical technique (*400) NF-kB p65 positive reaction material presents brown, the cell nucleus was dyed, the cell membrane and cytoplasm were not dyed

NF-kB p65 positive reaction material presents brown, the cell nucleus was dyed, the cell membrane and cytoplasm were not dyed, and the negative control was not dyed (Figure 4).

### The expression of NF-kB p65 comparing among the different group

The positive cell numbers of model group was more than the control group (*P <* 0.05), and the positive cell numbers of inhibitor group was less than model group (*P <* 0.05), nevertheless, there was no significant divergence between control group and inhibitor group (*p >* 0.05) (Table 2).

### The expressions of AQP1, AQP3, AQP8 in the colon by PCR technique

The expressions of AQP1, AQP3 and AQP8 mRNA of model group were less than the control group (*P <* 0.05), and the expressions of inhibitor group were more than model group (*P <* 0.05), however, there was no difference between control group and inhibitor group (*p >* 0.05) (Table 3).

**Table 3:** The expressions of AQP1, AQP3, AQP8 in the colon by RT-PCR technique

	Control group	Model group	Inhibitor group
AQP1	27.720 ± 0.841	24.511 ± 0.472*^a^*	26.776 ± 0.695*^b^*
AQP3	24.163 ± 1.027	20.814 ± 0.569*^a^*	23.492 ± 0.853*^b^*
AQP8	27.387 ± 0.850	23.438 ± 0.548*^a^*	26.007 ± 0.781*^b^*

^a^*P* < 0.05 compare with control group; ^b^*P* < 0.05 compared with model group.

### The expressions of AQP1, AQP3, AQP8 and NF-kB p65 in the colon by Western blot technique

**Figure 5:**
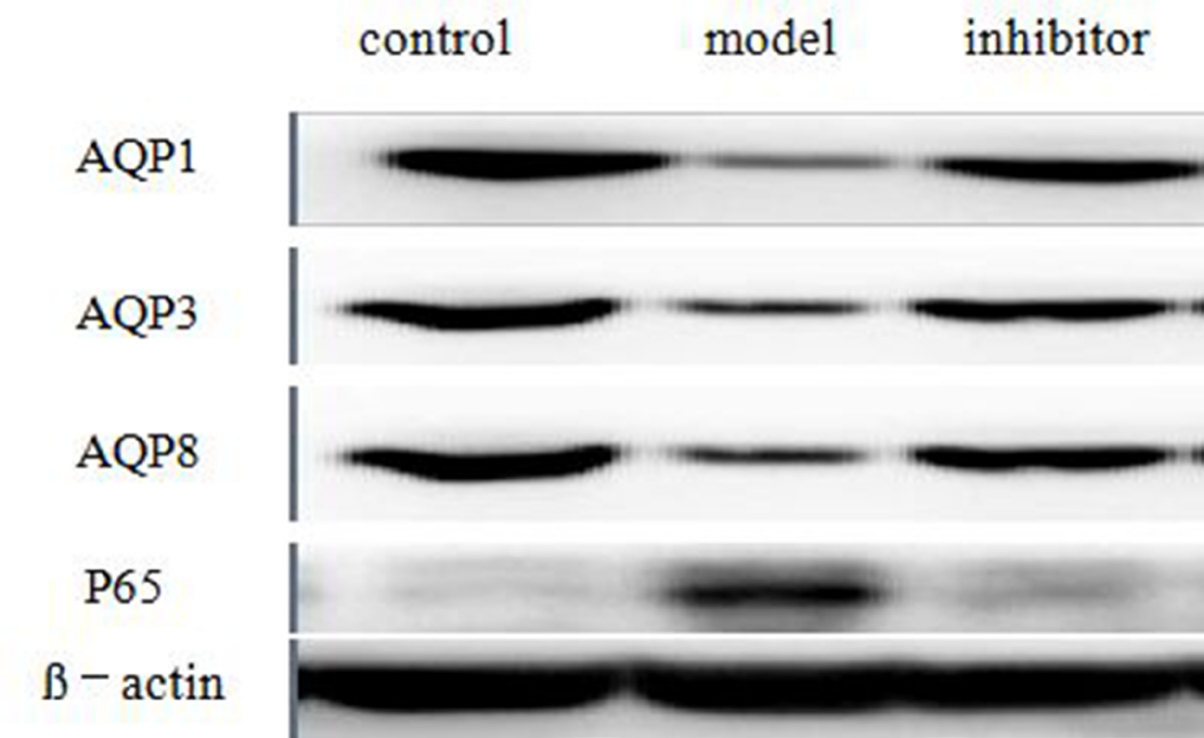
The expressions of AQP1, AQP3, AQP8 and NF-κB p65 in the colon by Western blot technique The expressions of AQP1, AQP3 and AQP8 protein of model group were less than the control group (*p* < 0.05), and the expressions of inhibitor group were more than model group (*p* < 0.05), The expressions of NF-κB p65 protein of model group were more than the control group (*p* < 0.05), and the expressions of inhibitor group were less than model group (*p* < 0.05).

The expressions of AQP1, AQP3 and AQP8 protein of model group were less than the control group (*P <* 0.05), and the expressions of inhibitor group were more than model group (*P <* 0.05), nevertheless, there was no significant divergence between control group and inhibitor group (*p >* 0.05). The expressions of NF-kB p65 protein of model group were more than the control group (*P <* 0.05), the expressions of inhibitor group were less than model group (*P <* 0.05), and the expression of inhibitor group was more than control group(*P <* 0.05). (Figure 5) (Table 4)

**Table 4:** The expressions of AQP1, AQP3, AQP8 and NF-κB p65 in the colon by Western bolt technique

	Control group	Model group	Inhibitor group
**AQP1**	29.325 ± 0.305	19.708 ± 0.744*^a^*	27.310 ± 0.985*^b^*
**AQP3**	29.263 ± 0.318	19.557 ± 0.944*^a^*	27.633 ± 0.683*^b^*
**AQP8**	28.647 ± 0.355	21.105 ± 0.621*^a^*	27.885 ± 1.716*^b^*
**P65**	13.210 ± 0.933	56.818 ± 2.834*^a^*	21.888 ± 1.237*^b^*

^a^*P* < 0.05 compare with control group; ^b^*P* < 0.05 compared with model group

## DISCUSSION

According to Rome IV criteria, the diagnostic criteria for Irritable bowel syndrome is defined as recurrent abdominal pain on average at least 1 day/week in the last 3 months, associated with 2 or more of the following:related to defecation, associated with a change in frequency or form (appearance) of stool [16]. IBS is a chronic biopsychological disorder which has complex symptoms appearing as altered bowel habits without organic pathology[17]. As we know, IBS is so common that it affects nearly 10–20% of adolescents and adults in western societies and is also the frequentest cause of children with recurrent abdominal pain [18, 19]. Several hypotheses have been proposed that the pathogenesis of IBS includes altered gut microbiota, visceral hypersensitivity, dysmotility, gastrointestinal infection and infestation, dysregulation of brain-gut axis, psychological and genetic factors [20]. Despite the growing body of literature, IBS pathophysiology remains poorly understood.

Nowadays, at least 13 types of AQPs (AQP0–12) have been identified in mammals. AQP0, AQP1, AQP2, AQP4, AQP5, AQP6 and AQP8 are reported be selectively permeable to water; and AQP3, AQP7, AQP9, AQP10 are permeable to water, glycerol and urea [21]. In mammals, AQPs are widely present in the digestive tract, including salivary gland, esophagus, stomach, small and large intestines, liver, gallbladder, bile duct and pancreas[22]. Currently several researches reported that AQPs play important roles in gastrointestinal diseases, however, there is few research reported about IBS. It isreported that several AQP types are found in gastrointestinal epithelia, with AQP1, 3, 7, 10 and 11 being the most abundantly expressed in the whole digest tract, and AQP4 and 8 are expressed selectively in the stomach and colon [23]. Several studies revealed that AQP1, AQP3 and AQP8 are closely connected with water transportation in colon [23, 24]. Another research showed that AQP-1 increases osmotic water permeability and locally facilitates the rapid, trans-membrane flux of water in plasma membrane blebs [25]. It is also reported that in the mediation of AQP3, the fecal water content in the colon is controlled by the transport of water from the luminal side to the vascular side[26]. The knockdown of AQP3 was reported to be connected with a decreasing of the expression of Claudin-1 and Occludin and the increasing of bacteria translocation, which revealed that AQP3 was associated with intestinal permeability. In our research, we found that the expressions of AQP1, AQP3 and AQP8 of model group were down-regulated comparing with the control group which suggested that liquid water metabolic abnormalities might be one of the mechanisms of IBS connecting with AQPs. On the other hand, as the connection of AQPs and Occludin expression, it is also revealed that tight junction or intestine permeability might be another mechanism of IBS regulated by AQPs.

NF-kB protein family include RelA (p65), Rel (c-Rel), RelB, NF-kB1 (p50), and NF-kB2 (p52), composed by a conserved Rel homology domain which is responsible for dimerization, nuclear localization and DNA-binding [27]. NF-kB pathway can be activated through two distinct signaling pathways: the classical pathway and the alternative pathway, and in the classical pathway, degradation of IkBa by IKKb activation triggers the translocation of various heterodimers, predominantly p65/p50, to the nucleus [28]. The heterodimer of NF-κB p65 and p50 subunit is a common form of NF-kB. NF-kB was also reported as an important pathway in the chronic intestinal inflammation [29]. That periostin mediates intestinal inflammation via the activation of NF-κB pathway, which suggests that periostin may be a potential therapeutic target for inflammatory bowel disease [30] has been reported. One latest study showed that the activation of TLR4 by NCI increase CBS expression, which is mediated by the NF-κB pathway, final lead to visceral hypersensitivity [31]. In our research, we found that NF-κB p65 of model group was up-regulated, and after the inhibitor of NF-κB pathway, the expression of NF-κB p65 was down-regulated again, which revealed NF-κB pathway played a role in the mechanism of IBS. And as NF-kB is a main regulator of inducing several genes including inflammatory and immune response, we suspected that the mechanism of NF-κB pathway for IBS might involve in the immune, inflammation and visceral hypersensitivity.

Until now we can't search the study about IBS connected with AQP and NF-κB pathway. There was a study found that once NF-κB activated by a hypertonic medium, can decreased AQP-2 mRNA and protein expression through binding of NF-κB complexes to specific kB elements of the AQP-2 promoter [32]. That IL-1β induces expression of AQP4 through a NF-kB pathway without involvement of denove protein synthesis in rat astrocytes has been reported [33]. Another study reported that lack of AQP5 showed declined activation of mitogen-activated protein kinase and NF-κB pathways in lungs before and after Pseudomonas aeruginosa(PA) infection [34]. In our research, the expression of AQP1, AQP3 and AQP8 was up-regulated after the injection of inhibitor of NF-κB pathway. Thus, our research revealed that liquid water metabolic abnormalities and intestine permeability might be the mechanism of IBS by regulating AQP1, AQP3 and AQP8 via NF-κB pathway. Worth attention, we found that the IBS model rats could not return to the normal state of the control group by inhibiting NF-κB.

## MATERIALS AND METHODS

### Subjects

There were 18 adult female SD big rats, the weight of every rat was about 200 g. Feeding environment was provided by experimental animal center of Zhejiang Chinese Medical University. 18 SD rats were divided randomly into three groups. The control group was 6, model group was 6, and inhibitor group was 6. They were put into the environment where the temperature was 22–24°C, the humidity < 60%, the noise < 50 db.

### Experimental procedure and methods

### Group

### Control group

They were normal rats. The control group was injected by 1 ml saline for control. After 2 weeks of normal eating and drinking, we observed the condition of rats. Then the rats’ visceral sensitivity was evaluated by abdominal withdraw reaction. The AWR scoring criteria are shown in Table 5 [35].

**Table 5:** Abdominal withdrawal reflex (AWR) scoring criteria

Score 0	No behavioral response to colorectal distension (CRD)
**Score 1**	Immobile during distension of colorectum (CR) and occasional appearance of brief head motion after a pause at the onset of the stimulation
**Score 2**	A mild contraction of abdominal muscles, but no lifting of abdomen off the plattorm
**Score 3**	A strong contraction of abdominal muscles and lifting of abdomen off the platform, no lifting of pelvic structure off the platform
**Score 4**	Arching body and lifting of pelvic structure and scrotum

### Model group

The conditioned stimulus was camphor ball special odor. The unconditioned stimulus was rectal distention pressure (> 60 mmHg (1 mmHg = 0.133 kPa)) combining with extremities constraint. Rats were put into the cage with camphor ball in it, we fixed the extremities and trunk of the rats for 45 min. At this time inserted catheter into rectum. The distance from air ballon distal end to anal was about 1 cm. The catheter was fixed at the root of the tail. The balloon volume was 1.6 ml (hydrostatic pressure in Ballon's space > 60 mmHg) and lasted for 60 s, intermittent exhaust 3 mins and filled gas 10 times once. This was a stress process. There was one process in the first day, the same process was performed on the second day at the same time. The conditioned stimulus Without the unconditioned stimulus was done on the fourth day. The completed process was repeated once on the fifth day. The process same as the fourth day was performed on the sixth day. The conditioned stimulus was performed on the eighth day [36, 37]. Then the rats’ visceral sensitivity was evaluated by abdominal withdraw reaction.

### Inhibitor group

The IBS model rats were injected into the abdominal cavity with the inhibitor of NF-kB (PDTC, 50 mg/kg/d) once after completing the models.

### Model authentication

Visceral sensitivity was evaluated by abdominal withdraw reaction (AWR). 8 F urethral catheter which was lubricated by liquid paraffin was inserted per anum. The distance from air ballon distal end to anal was about 1 cm, and it was fixed at the root of the tail. The rats were put on the platform, after they accommodated the environment, we gradually affused water into sacculus, and recorded the water injection rate when the rats raised the abdomen and made the back like a bow when AWR score was 3. Rectal distention lasted for 30 s every time, and repeated 3 times. And then we took the mean number.

### Experimental sample

After a laparotomy incision, a portion of the colon was removed and placed in an oxygenated Tyrode's solution. A segment of 2 cm length colon were mounted in a 10ml organ bath containing Tyrode's solution that was bubbled with a 95% O2 and 5% CO2 mixture, and the temperature was held at 37°C.

### Experimental procedure (immunohistochemical technique)

### Two footwork

We dropwised 3% hydrogen dioxide on tissue away from light and incubated for 15min. And then the tissue was flushed by distilled water, and we put the chips into PBS balanced solution, and soaked for 5 min, for 3 times. AG dark was repaired. We dropped 50–100 ml antibody fluid on the tissue, and incubated for 30 min in the ambient temperature. Then we washed chips with PBS, and soaked the chips in PBS balanced solution for 4 min 3 times. Then we dropped appreciable proportion diluted biotin labeling antibody (1%BSA-PBS to dilute), and incubated for 30 min in the ambient temperature. After that we washed the chips with PBS, and soaked them in the PBS balanced solution for 5 min 3 times. Then we dropped 50–100 ml developer DAB fluid, and incubated for 5–20 min, after coloration completely, and then the tissue was washed by distilled water. The tissue was dewatered by 85%, 90%, 95%, 100%, 100%, 100% alcohol. Then we put the chips into xylene for 5 min, 3 times.

### Negative control

Replaced one antibody with PBS, the consequence was negative.

### Analytical method

We used the computer image analysis software (the Carl Zeiss of the Imaging Systems of the Carl Zeiss company) to analyze images. The images were put under 40 times object glass. We found the typical places, and took 10 high power campus visualis (×400) successively. Then we analyzed the masculine expression by quantitative analysis and calculated photodensity.

### Experimental procedure (RT-PCR technique)

Total RNA was extracted from duodenal tissues with Trizol (Invitrogen, Gaithersburg, MD, USA) using the one-step method. After purification, RNA concentration was analyzed using Nanodrop (Nanodrop Technologies, Wilmington, DE) and quality testing was conducted using BioAnalyzer (Agilent Technologies, Palo Alto, CA). After preparing total RNA, we did primer test and sample assay. Then we performed reverse transcription. After creating and setting up a plate document, we prepared the PCR reaction plate, after that we run the PCR reaction plate, then we analyzed the result. he main procedure was high-throughput sequencing on independent samples. Small RNA was purified from total RNA to enrich molecules in the range of 16–30 nt, and then 3′ and 5′ linker sequences were attached, and SuperScript II reverse transcriptase was used to synthesize cDNA. PCR amplification was conducted.

### Experimental procedure (Western blot technique)

Protein measurements were performed on the prepared cell extracts and 7.5% SDS gel was also prepared. After the polymerization of the gel, the cell extracts were diluted in the sample buffer and boiled at 95°C for 5 min. With the samples executed in the gel, proteins were transferred into membranes by the use of semidry transfer method. The transfer will be realized at 100 V within 1 h. It was held in PBS solution (blocking solution) with 5% milk powder and 0.1% tween 20. The membrane will be incubated with primer antibodies overnight. And the membrane was washed 3 times using PBS solution with 0.1% Tween 20 for 5 min and second antibody application was performed. After membrane incubation with rabbit second antibody for 1 h, it was again washed 3 times using PBS solution with 0.1% Tween 20 for 5 min. ECL solution was used with the purpose of viewing and filming proteins in the membrane. Horseradish peroxidase enzyme linked to the second antibody catalyzes Lumigan PS-3 substrate in ECL solution.

### Statistical analysis

The data was demonstrated as`x ± SE. We used SPSS 17.0 software as statistical method. evene's test for equality of variances, differences between three groups were compared using one-factor analysis of variance (ANOVA), tatistical significance was taken as *P <* 0.05.

## CONCLUSIONS

As a chronic biopsychological disorder, IBS is characterized by altered bowel habits excluding organic disorders. However, the real mechanism of IBS remains unknown. Our research showed that liquid water metabolic abnormalities and intestine permeability Alteration might be the mechanism of IBS by down-regulating AQP1, AQP3 and AQP8 via NF-κB pathway.
